# Preoperative Hill's classification as a predictor of postoperative gastroesophageal reflux disease following laparoscopic sleeve gastrectomy: a prospective multicenter study from Egypt

**DOI:** 10.1186/s12893-026-03531-w

**Published:** 2026-02-12

**Authors:** Atteyat A. Semeya, Esayed Elmokadem, Raafat S. A. Abdel Hafez, Mohammed Siam, Amira A. A. Othman, Mohamed Mahmoud Abdalgaliel

**Affiliations:** 1 Hepatology, Gastroenterology and Infectious Diseases Department, Benha Teaching Hospital, Benha, Egypt; 2 General Surgery Department, Benha Teaching Hospital, Benha, Egypt; 3 Internal Medicine Department, Damanhour Medical National Institute, Damanhour, El Beheira Egypt; 4 General Surgery Department, Faculty of Medicine, Suez University, Suez, Egypt; 5 Internal Medicine Department, Faculty of Medicine, Suez University, Suez, 43511 Egypt; 6 General Surgery Department, Damnhour Medical National Institute, Damanhour, El Beheira Egypt

**Keywords:** Bariatric surgery, Sleeve gastrectomy, Gastroesophageal reflux, Hill classification, Endoscopy, Egypt

## Abstract

**Background:**

Laparoscopic sleeve gastrectomy (LSG) is the most widely used bariatric procedure, but its influence on gastroesophageal reflux disease (GERD), whether inducing, aggravating, or improving it, remains a subject of ongoing debate. This study evaluated whether preoperative Hill’s classification can predict postoperative GERD and identified other endoscopic factors related to this outcome in an Egyptian multicenter cohort.

**Methods:**

In this prospective multicenter cohort, 106 consecutive morbidly obese patients undergoing primary LSG (January 2023–June 2024) were evaluated. All underwent standardized preoperative esophagogastroduodenoscopy (EGD) with Hill, Los Angeles (LA), and Z-line appearance (ZAP) grading; postoperative assessments (GerdQ and repeat EGD) were performed at 6 months and 1 year. GERD was defined as erosive esophagitis (LA ≥ B) on follow-up EGD, LA Grade A esophagitis with concurrent pathological acid exposure (DeMeester score > 14.7), or, in symptomatic patients with normal endoscopy, pathological acid exposure alone. Ambulatory 24-h pH monitoring (off PPI therapy) was performed preoperatively in all patients and postoperatively only in symptomatic patients with a normal endoscopy. Follow-up was complete for all 106 patients at 1 year.

**Results:**

At 1 year, 39/106 patients (36.8%) had objective GERD, including a 26.0% incidence of de novo GERD among those without preoperative evidence of the condition. Preoperative Hill Grades III–IV were strongly associated with postoperative GERD in the primary analysis and remained independently predictive after adjustment for age, sex, BMI, and %EWL (aOR 3.92; 95% CI 1.98–7.76; *p <* 0.001). This association remained robust in a sensitivity analysis applying a stricter, contemporary GERD definition (aOR 3.71; 95% CI 1.82–7.55; *p <* 0.001). Time-to-event analysis showed reduced GERD-free survival in patients with Hill III–IV (1-year GERD-free survival 41.9% vs 71.4% for Hill I/II; Cox HR 3.60, 95% CI 1.85–7.00; log-rank *p <* 0.001). Preoperative esophagitis (LA ≥ A) and distorted Z-line (ZAP ≥ II) were associated with GERD in univariate analyses, but Hill grade was the only robust independent predictor. A Random Forest classifier incorporating Hill grade, hiatal hernia, and HbA1c achieved an internal AUC of 0.84 (95% CI 0.76–0.91), outperforming logistic regression (AUC 0.76; *p =* 0.03).

**Conclusion:**

Preoperative Hill’s grading of the gastroesophageal flap valve (GEFV) independently predicts GERD after LSG. Its prognostic value persists after adjusting for hiatal hernia and is consistent under contemporary diagnostic criteria. Concurrent cruroplasty does not completely offset the risk of a defective valve. Routine preoperative EGD with Hill assessment improves risk stratification and may guide individualized surgical planning, including consideration of RYGB in high-risk cases. This is the first multicenter Egyptian study to apply Hill grading together with machine-learning methods for reflux prediction.

**Trial registration:**

Not applicable.

## Introduction

The escalating global obesity epidemic has established itself as a principal driver of gastroesophageal reflux disease (GERD), with rising obesity rates closely linked to a marked increase in GERD occurrence [1]. In Egypt, obesity prevalence parallels the worldwide pattern; recent estimates indicate that more than 35% of adults are obese, creating a substantial strain on healthcare services [2, 3]. This epidemic contributes to major morbidity and mortality through cardiovascular diseases, osteoarthritis, diabetes, cancer, and GERD itself [2]. Supporting this, a meta-analysis by Hampel et al. [1] confirmed a consistent, positive association between excess body weight and the prevalence and severity of GERD. This relationship may be further influenced in Egypt by local dietary habits and lifestyle factors.

For severe obesity, bariatric surgery provides the most durable long-term control of weight and associated comorbidities [3, 4]. Within Egypt, surgical management of obesity has expanded rapidly, with laparoscopic sleeve gastrectomy (LSG) becoming the dominant procedure, favored for its favorable balance of procedural efficacy and technical feasibility. Among the available options, which include gastric banding and Roux-en-Y gastric bypass (RYGB), LSG stands out. Initially introduced as the first stage of a biliopancreatic diversion with duodenal switch, it is now firmly established as a definitive stand-alone operation [5]. The procedure's design, which maintains gastrointestinal continuity without intestinal bypass or anastomosis, also allows for straightforward endoscopic evaluation postoperatively [6].

A high prevalence of GERD symptoms, affecting nearly half of morbidly obese patients, underscores the strong link between obesity and reflux. The underlying pathophysiology is largely mechanical, where increased intra-abdominal pressure stresses the esophagogastric junction (EGJ), fosters transient lower esophageal sphincter (LES) relaxations, and elevates the risk of hiatal hernia (HH), a key contributor to reflux. The presence of a hiatal hernia compromises the gastroesophageal flap valve (GEFV), disrupting the normal pressure dynamics at the LES and facilitating the reflux of gastric contents [7]. In a recent meta-analysis, Yeung et al. pooled data from 46 studies (10,718 patients) undergoing LSG and reported a 19% rise in GERD symptoms postoperatively. The incidence of de novo GERD was 23%, with postoperative esophagitis in 30%, Barrett’s esophagus in 6%, HH in 41%, proton-pump inhibitor use in 38%, and a 4% conversion rate to Roux-en-Y gastric bypass (RYGB) for severe reflux [8]. These findings emphasize the importance of preoperative risk stratification, particularly in Egypt, where environmental and genetic factors may modify GERD behavior. Early identification of patients at risk of postoperative reflux could optimize procedure selection and reduce the need for later revisional surgery.

The present study introduces a novel contribution through its prospective, multicenter design and systematic use of Hill’s, Los Angeles (LA), and Z-line Appearance (ZAP) classifications to establish a detailed preoperative endoscopic profile. Hill’s classification provides a reproducible method for assessing the gastroesophageal flap valve (GEFV), a major structural component of the anti-reflux barrier [9, 10]. High Hill grades (III–IV) are closely associated with GERD severity, reduced LES pressure, and the presence of hiatal hernia [11]. Despite this, the role of Hill’s classification in predicting postoperative reflux after LSG has not been thoroughly examined, especially within Middle Eastern and North African (MENA) populations. Most available studies are retrospective or lack standardized prospective endoscopic follow-up, limiting their clinical utility [12].

Despite accumulating reports, there is a lack of prospective, multicenter studies that apply standardized endoscopic grading (Hill, LA, ZAP) with structured longitudinal endoscopic follow-up after LSG, especially in the Middle East & North Africa (MENA) region. We hypothesized that higher preoperative Hill grades (III–IV) independently predict the development of objective GERD within one year after laparoscopic sleeve gastrectomy, even after adjustment for demographic, metabolic, and anatomical confounders. The primary aim of this prospective multicenter study was to determine whether preoperative Hill’s classification predicts postoperative GERD. Secondary aims included assessing associations between preoperative LA and ZAP grades and postoperative GERD, and correlating endoscopic findings with symptom trajectories and quality of life over one year.

## Subjects and methods

### Study population and design

This prospective, multicenter, observational cohort study was conducted across collaborating clinical sites in the Nile Delta region of Egypt. The study was anchored at the Endoscopy Unit of the Hepatogastroenterology Department at Banha Teaching Hospital, with additional patient recruitment and surgical procedures performed at a high-volume private bariatric center in the region. The research team included specialists from Banha Teaching Hospital and faculty members from the Internal Medicine and General Surgery departments of Suez University, fostering a collaborative, multidisciplinary environment. Patient enrollment and follow-up spanned from January 2023 to June 2024. The study was designed and reported in accordance with the STROBE (Strengthening the Reporting of Observational Studies in Epidemiology) guidelines to ensure methodological rigor. This multicenter framework improves the applicability of our findings across different healthcare settings in Egypt.

A formal sample size calculation was performed a priori using G*Power statistical software (version 3.1.9.7). The calculation was based on the primary objective of detecting a significant difference in the incidence of postoperative GERD between patients with a competent gastroesophageal flap valve (Hill Grades I-II) and those with an incompetent valve (Hill Grades III-IV). Drawing upon preliminary data and existing literature, we anticipated GERD incidence rates of 15% and 45% in the two groups, respectively [11]. At 1-year follow-up, the observed incidences of GERD (17.5% in Hill I–II vs. 51.6% in Hill III–IV) closely approximated these assumptions, confirming that the study was adequately powered to detect the prespecified effect size. To detect this effect size with a statistical power of 90% (1-β = 0.90) and a two-sided Type I error rate (α) of 0.05, a minimum of 44 patients per Hill grade group (I–II vs III–IV) was required. To allow for attrition and ensure adequate power to detect the prespecified difference, the final target enrollment was set at 106 participants. A sensitivity analysis confirmed adequacy across plausible effect sizes.

### Ethical considerations

Before the initiation of any study-related procedures, the full research protocol was submitted for review and received formal approval from the Ethics Unit of Benha Teaching Hospitals, Egypt, Approval #: HB-000127, and was approved by the research ethics committees of all participating hospitals and the Egyptian Ministry of Health. This study was conducted in strict adherence to the ethical principles for medical research involving human subjects as outlined in the Declaration of Helsinki. A fundamental prerequisite for enrollment was the procurement of written, informed consent from every participant. This consent process involved a comprehensive discussion detailing the nature of the laparoscopic sleeve gastrectomy procedure, the specifics of the endoscopic evaluations, potential risks and benefits, the voluntary nature of participation, and the confidentiality of their data. An independent data safety monitoring board was established to oversee ethical compliance throughout the study.

### Inclusion and exclusion criteria

#### Inclusion criteria

The study cohort was derived from consecutive adult patients presenting with morbid obesity who were deemed suitable candidates for a primary LSG. To be eligible for inclusion, patients had to be between 18 and 60 years of age and have a Body Mass Index (BMI) of either ≥ 40 kg/m^2^ or ≥ 35 kg/m^2^ accompanied by at least one significant obesity-related comorbidity, such as type 2 diabetes mellitus, hypertension, or obstructive sleep apnea.

#### Exclusion criteria

A stringent set of exclusion criteria was applied to minimize confounding variables and ensure patient safety. Individuals were excluded from participation if they had a history of any previous upper gastrointestinal surgery, which could alter anatomical relationships and the interpretation of reflux mechanisms. Additional exclusion criteria encompassed any contraindication to undergoing an upper endoscopy, such as a severe coagulopathy or unstable cardiopulmonary status; current pregnancy or lactation; a preoperative endoscopic diagnosis of Barrett's esophagus or esophageal adenocarcinoma; the presence of a significant, uncontrolled psychiatric disorder that could compromise adherence to the follow-up protocol; an intraoperative conversion from the planned sleeve gastrectomy to an alternative procedure like Roux-en-Y Gastric Bypass; and finally, any patient lost to follow-up before completing the critical one-year endoscopic assessment. Patients with incomplete preoperative data were excluded to ensure data integrity.

### Preoperative evaluation protocol

All candidates underwent an extensive and standardized preoperative assessment. This began with a detailed clinical evaluation, including a full medical history and physical examination, with specific attention to anthropometric measurements (weight, height, and calculated BMI) and documented comorbidities.

Laboratory investigations formed a key part of the workup, encompassing a complete blood count, fasting blood glucose, glycated hemoglobin (HbA1c), liver function tests (AST, ALT), renal function tests (serum creatinine), serum albumin, and a coagulation profile (International Normalized Ratio, INR).

GERD-related symptomatology was quantitatively assessed using the validated Gastroesophageal Reflux Disease Questionnaire (GerdQ). A score of 8 or higher on this instrument was considered indicative of clinically significant GERD. For this study, the diagnosis of objective GERD was based on a hierarchical, multi-modal definition to ensure rigor: (1) endoscopic evidence of erosive esophagitis (Los Angeles grade B or higher) on follow-up EGD; or (2) LA grade A esophagitis with concurrent pathological acid exposure (DeMeester score > 14.7) on 24-h pH monitoring; or (3) in symptomatic patients with normal endoscopy, pathological acid exposure alone (DeMeester score > 14.7). The GerdQ questionnaire was thus used to quantify symptom burden and identify symptomatic patients for selective pH testing, but a score ≥ 8 was not sufficient for GERD diagnosis without objective endoscopic or pH-metric confirmation. This approach aligns with contemporary diagnostic standards, including the 2022 ACG Clinical Guideline.

**Fig. 1:**
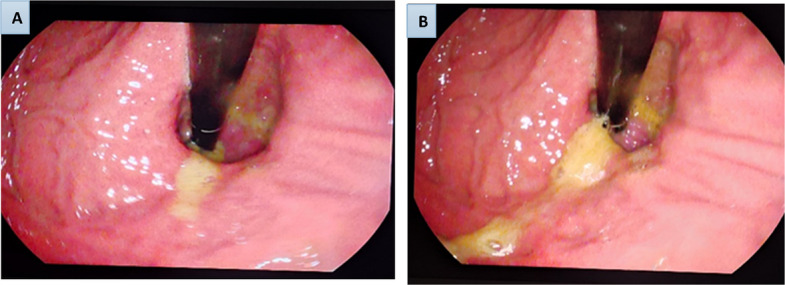
Preoperative endoscopic retroflexed view of the gastroesophageal junction demonstrating Hill’s classification grades III and IV. **A** Hill Grade III valve showing a distinctly gaping cardia with partial loss of the muscular ridge. The endoscope tip is seen entering the gastroesophageal junction with visible gastric folds not tightly opposed, indicating decreased lower esophageal sphincter competence. **B** Hill Grade IV valve demonstrating a wide-open gastroesophageal junction with complete loss of the valve fold and visible retroflexion of gastric mucosa around the scope. The endoscope shaft is fully exposed, consistent with severe valve incompetence and advanced reflux barrier disruption. These images represent endoscopic assessment of valve morphology and were obtained during preoperative esophagogastroduodenoscopy (EGD)

The cornerstone of the preoperative evaluation was the standardized esophagogastroduodenoscopy (EGD). All endoscopic procedures were performed by one of three dedicated endoscopists, each with extensive experience exceeding 500 procedures, who were blinded to the patients' clinical symptom scores. The EGD followed a strict, pre-defined protocol to ensure consistency. The gastroesophageal junction was meticulously evaluated, and the gastroesophageal flap valve was classified according to the established Hill grading system (I-IV) (Fig. 1A, B). The esophagus was inspected for the presence of erosive esophagitis, which was classified using the Los Angeles (LA) system. The configuration of the squamocolumnar junction (Z-line) was documented using the ZAP classification. The presence and size (in centimeters) of any hiatal hernia were also recorded. To obtain histopathological correlation, targeted biopsies were taken from the Z-line. These tissue samples were processed, embedded in paraffin, and stained with Hematoxylin and Eosin (H&E) as well as Periodic Acid–Schiff (PAS) reagent. All histopathological slides were reviewed by an experienced gastrointestinal pathologist who was blinded to the endoscopic findings, with a focus on identifying features of esophagitis and intestinal metaplasia. Inter-observer agreement for endoscopic grading (Hill, LA, ZAP) was assessed using Fleiss’ kappa for multiple raters; the kappa statistics (with 95% CIs) are reported in the Results section. Additionally, endoscopic images were systematically recorded, and a subset was reviewed by all three endoscopists to validate Hill's, LA, and ZAP classifications, with histopathological findings correlated to endoscopic grades to assess diagnostic concordance.

### Surgical procedure

**Fig. 2:**
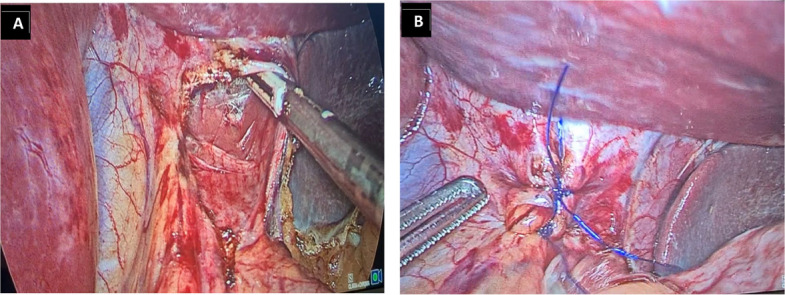
Intraoperative steps of posterior cruroplasty during laparoscopic sleeve gastrectomy in patients with hiatal hernia > 2 cm. **A** Dissection of the esophageal hiatus and identification of the right and left crura. **B** Posterior cruroplasty performed with interrupted non-absorbable sutures to restore diaphragmatic hiatus integrity

The laparoscopic sleeve gastrectomy was performed following a standardized technique by experienced bariatric surgeons at both centers, consistent with previously described methods [13, 14]. In brief, after creation of pneumoperitoneum, the greater curvature was mobilized with division of the short gastric vessels, and a 36-Fr bougie was positioned along the lesser curvature to calibrate the sleeve. Gastric transection was initiated approximately 4–6 cm proximal to the pylorus and advanced toward the angle of His using sequential linear stapler firings. The esophageal hiatus was routinely inspected; when a hiatal defect larger than 2 cm was identified, posterior cruroplasty was performed with interrupted non-absorbable sutures (Fig. 2A and B). Staple-line integrity was assessed using methylene blue instillation or air insufflation under saline, and intraoperative endoscopy was performed selectively to confirm the completeness and configuration of the staple line. Endoscopic images used for preoperative valve assessment (Fig. 1) should not be interpreted as representing surgical hiatal repair, which is illustrated in Fig. 2.

### Postoperative follow-up and outcome measures

Patients were systematically followed at two key intervals: 6 months and 1 year after their surgery. Each follow-up visit included a clinical reassessment with measurement of weight and BMI, administration of the GerdQ questionnaire, and a repeat esophagogastroduodenoscopy. This postoperative EGD adhered to the same rigorous, structured protocol as the preoperative examination, including the application of the Hill, LA, and ZAP classifications and procurement of biopsies for histology. Quality of life was evaluated using the Short Form-36 (SF-36) questionnaire to correlate GERD severity with patient-reported outcomes [15].

Ambulatory 24-h pH monitoring (wireless Bravo system or catheter-based system, according to site availability) was performed preoperatively in all participants after a 7-day washout from proton-pump inhibitors (PPIs) to quantify baseline esophageal acid exposure without pharmacological suppression. Postoperatively, it was performed selectively in symptomatic patients who had a normal follow-up endoscopy to objectively confirm pathological reflux. A DeMeester score > 14.7 was considered diagnostic of pathological acid exposure.

The primary outcome measure for this study was the development of objective gastroesophageal reflux disease (GERD) within the first postoperative year. Objective GERD was defined as the presence of erosive esophagitis, Los Angeles (LA) grade B or higher on follow-up endoscopy, or LA grade A with concurrent pathological acid exposure (DeMeester score > 14.7) on 24-h ambulatory pH monitoring, or, in symptomatic patients with normal endoscopy, a pathological DeMeester score > 14.7 alone. This definition aligns with the 2022 ACG Clinical Guideline, which indicates that LA grade A esophagitis alone is insufficient for GERD diagnosis without abnormal reflux monitoring.

Secondary outcome measures included the longitudinal change in the Hill's classification grade, the specific incidence of de novo GERD in patients without preoperative evidence of the condition, the correlation between preoperative endoscopic findings and postoperative symptom scores, the rate of surgical conversion to Roux-en-Y Gastric Bypass for severe, medically refractory GERD, and the proportion of patients requiring daily proton-pump inhibitor therapy at the one-year mark.

### Statistical analysis

Data analysis was performed using the IBM SPSS Statistics software package, version 28.0, supplemented with R software (version 4.2.1) for advanced modeling. The normality of continuous variables was assessed with the Shapiro–Wilk test, with normally distributed data presented as mean ± standard deviation (SD) and non-normally distributed data as median and interquartile range (IQR). Categorical variables are expressed as frequencies and percentages. For comparative analyses, baseline characteristics were compared using Student's t-test, Mann–Whitney U test, or Chi-square/Fisher's exact test, as appropriate, while ANOVA with post-hoc Tukey testing was applied for multi-group comparisons.

Predictors of the primary outcome were first identified through univariate logistic regression, with variables significant at *p <* 0.10 entered into a multivariate logistic regression model using stepwise selection to ascertain independent predictors, reported as odds ratios (OR) with 95% confidence intervals (CI). Model fit was evaluated with the Hosmer–Lemeshow test and Nagelkerke R^2^. Time-to-event analysis was conducted using Kaplan–Meier curves with log-rank testing, and Cox proportional hazards models were used to calculate hazard ratios (HR), adjusting for confounders such as age, sex, BMI, and percentage excess weight loss.

To provide deeper insights, advanced analyses were implemented: a Random Forest classifier was trained to predict 1-year postoperative GERD, with the dataset randomly split into training (70%) and held-out test (30%) sets stratified by outcome; model building and tuning were performed in R (randomForest and caret packages) with hyperparameters tuned via fivefold cross-validation on the training set; the final model used 500 trees and an optimized mtry value, with performance assessed on the held-out test set and reported as area under the ROC curve (AUC), sensitivity, specificity and calibration plots; AUCs were compared using DeLong's test and variable importance was reported using Mean Decrease in Gini; model calibration was assessed with the Brier score and visually with calibration plots, generated using the caret and pROC packages in R. Latent Class Mixed Models (LCMM) identified distinct GERD symptom trajectories based on Bayesian Information Criterion; interaction effects were tested using multiplicative terms in logistic regression; restricted cubic spline regression characterized the non-linear dose–response relationship between Hill's grade and GERD risk; and proportional odds ordinal logistic regression assessed associations with esophagitis severity. Correlations between ordinal endoscopic scores were quantified with Spearman's ρ, and inter-observer agreement was assessed with Fleiss' Kappa. Missing data (affecting < 5% of observations across variables with missingness) were handled using multiple imputation by chained equations (MICE) with m = 20 imputations in R (mice package), and pooled estimates were combined according to Rubin's rules. A two-tailed *p*-value < 0.05 defined statistical significance for all analyses.

## Results

### Baseline characteristics of a high-risk cohort with impaired quality of life

The study enrolled 106 patients with a mean age of 38.5 ± 11.2 years, predominantly female (72.6%), and a mean preoperative BMI of 46.2 ± 5.8 kg/m^2^ (Table 1). Comorbidities were prevalent, with hypertension (48.1%) and type 2 diabetes (40.6%) being the most common. The mean preoperative GerdQ symptom score was 7.2 ± 3.1, with 45.3% of patients scoring ≥ 8, indicating a substantial baseline burden of reflux symptoms.

**Table 1:** Baseline demographic, clinical, endoscopic, and quality of life characteristics

Characteristic	Total Cohort (*n =* 106)	No GERD at 1 Year (*n =* 67)	GERD at 1 Year (*n =* 39)	*p*-value
**Demographics**				
Age (years), mean ± SD	38.5 ± 11.2	37.8 ± 10.9	39.8 ± 11.7	0.351
Sex, n (%) Female	77 (72.6%)	52 (77.6%)	25 (64.1%)	0.127
**Anthropometrics**				
BMI (kg/m^2^), mean ± SD	46.2 ± 5.8	45.9 ± 5.5	46.8 ± 6.3	0.421
**Clinical & Symptomatic Measures**				
Preop GerdQ score, mean ± SD	7.2 ± 3.1	6.8 ± 2.9	7.9 ± 3.3	0.089
GerdQ ≥ 8, n (%)	48 (45.3%)	27 (40.3%)	21 (53.8%)	0.172
**Preoperative Endoscopic Findings**				
Hill's Grade III/IV, n (%)	62 (58.5%)	29 (43.3%)	33 (87.2%)	< 0.001
**LA Grade, n (%)**				0.089*
None	73 (68.9%)	50 (74.6%)	23 (59.0%)	
Grade A	20 (18.9%)	11 (16.4%)	9 (23.1%)	
Grade B	11 (10.4%)	5 (7.5%)	6 (15.4%)	
Grade C/D	2 (1.9%)	1 (1.5%)	1 (2.6%)	
Hiatal Hernia, n (%)	40 (37.7%)	19 (28.4%)	21 (53.8%)	0.008
**Quality of Life (SF-36)**				
Physical Component Score (PCS)	38.4 ± 7.2	39.8 ± 6.8	35.9 ± 7.4	0.015
Mental Component Score (MCS)	41.6 ± 7.8	42.5 ± 7.5	40.0 ± 8.1	0.104

All descriptive and comparative analyses for this table used pooled estimates from multiple imputation (m = 20). Percentages are based on the imputed dataset; results were materially unchanged in complete-case sensitivity analyses. Data are presented as mean ± SD or n (%). *SD* Standard deviation, *BMI* Body mass index, *LA* Los Angeles, *p*-values from t-test, Mann–Whitney U, or Chi-square test comparing GERD vs. non-GERD groups. For key comparisons, between-group differences are available on request, including mean differences (95% CI) for continuous variables and odds ratios (95% CI) for categorical variables.* Overall p-value for LA Grade (None vs. Any) = 0.089; Chi-square test for trend across severity grades (None → A → B → C/D) was not significant (*p =* 0.102)

The systematic preoperative endoscopic evaluation revealed a critical finding: a majority of patients (58.5%) presented with a compromised gastroesophageal flap valve (Hill Grade III or IV). When examining baseline esophagitis severity, 20 patients (18.9%) had LA Grade A, 11 (10.4%) had Grade B, and 2 (1.9%) had Grade C/D, while 73 patients (68.9%) had no erosive esophagitis (Table 1). Notably, baseline quality of life was significantly impaired, with a mean SF-36 Physical Component Score (PCS) of 38.4 ± 7.2, reflecting the substantial burden of morbid obesity. Among patients with hiatal hernia (*n =* 40, 37.7%), 38 (95.0%) underwent concurrent cruroplasty during sleeve gastrectomy.

A comparison of baseline characteristics showed that patients who later developed GERD had a significantly higher prevalence of pathological Hill's grades (87.2% vs. 43.3%, *p <* 0.001) and a lower baseline PCS (35.9 vs. 39.8, *p =* 0.015). The presence of hiatal hernia (37.7%) further stratified risk, with a higher incidence in the GERD group (53.8% vs. 28.4%, *p =* 0.008), suggesting a mechanical contribution to baseline risk. While preoperative GerdQ scores were numerically higher in the group that later developed GERD (7.9 vs. 6.8), this difference did not reach statistical significance (*p =* 0.089), underscoring the limited predictive value of symptoms alone compared to objective endoscopic anatomy.

Inter-observer agreement for endoscopic grading was as follows (Fleiss’ κ, 95% CI): Hill classification κ = 0.82 (95% CI 0.76–0.88), Los Angeles (LA) classification κ = 0.78 (95% CI 0.71–0.85), ZAP classification κ = 0.75 (95% CI 0.68–0.82). These κ values indicate substantial agreement among the three endoscopists.

Histopathological analysis of Z-line biopsies was performed for all patients. Preoperatively, no cases of intestinal metaplasia or Barrett's esophagus were identified, which was consistent with the study's exclusion criteria. At the one-year follow-up, biopsies remained negative for intestinal metaplasia in all 106 patients. Furthermore, the presence of histologic esophagitis on biopsy showed a significant correlation with the severity of the endoscopic Los Angeles (LA) grade (*p <* 0.001).

### Postoperative metabolic improvements and endoscopic progression

At one year, 39/106 patients (36.8%) met the predefined objective criteria for GERD: 27 based on erosive esophagitis (LA grade ≥ B) and 12 based on pathological 24-h pH monitoring alone (DeMeester score > 14.7) in symptomatic patients with normal endoscopy or LA grade A esophagitis. Preoperatively, all 106 patients underwent pH monitoring; the mean DeMeester score was 18.4 ± 12.1, with 44 patients (41.5%) having pathological acid exposure. Postoperatively, pH monitoring was performed in 28 symptomatic patients with normal endoscopy or LA grade A, of whom 18 (64.3%) had pathological acid exposure (mean DeMeester score 24.6 ± 10.8). Among the 73 patients without objective preoperative GERD, 19 developed de novo GERD (26.0%), all confirmed by endoscopic or pH-metric evidence. Clinically, 31 patients (29.2%) required daily proton-pump inhibitor (PPI) therapy at one year, and 3 patients (2.8%) underwent conversion to Roux-en-Y gastric bypass (RYGB) for severe, medically refractory GERD (Table 2).

**Table 2:** Longitudinal Metabolic, Endoscopic, pH-Metric, and Clinical Outcomes

Parameter	Preoperative (*n =* 106)	6 Months Postop (*n =* 106)	1 Year Postop (*n =* 106)	*p*-value (Preop vs. 1 Year)
**Laboratory Investigations**				
FBG (mg/dL), mean ± SD	112.4 ± 60.1	91.8 ± 19.5	88.3 ± 16.2	< 0.001
HbA1c (%), mean ± SD	5.9 ± 1.1	4.7 ± 0.6	4.8 ± 0.5	< 0.001
**Endoscopic Findings**				
LA Grade C/D, n (%)	2 (1.9%)	6 (5.7%)	5 (4.7%)	0.041
Hill's Grade III/IV, n (%)	62 (58.5%)	60 (56.6%)	65 (61.3%)	0.720
ZAP Grade ≥ II, n (%)	31 (29.2%)	30 (28.3%)	34 (32.1%)	0.610
**pH-Metric Findings**				
DeMeester score, mean ± SD	18.4 ± 12.1	–	15.2 ± 11.4*	0.038
Pathological acid exposure (DeMeester > 14.7), n (%)	44 (41.5%)	–	18 (17.0%)*	< 0.001
**Medical & Surgical Outcomes**				
Daily PPI use, n (%)	22 (20.8%)†	28 (26.4%)	31 (29.2%)	0.112
Conversion to RYGB, n (%)	–	1 (0.9%)	3 (2.8%)	–

Data are presented as mean ± SD or n (%). *SD* Standard deviation, *FBG* Fasting blood glucose, *HbA1c* glycated hemoglobin, LA = Los Angeles, *ZAP* Z-line appearance; p-values from paired t-test or McNemar’s test. * Postoperative pH monitoring was performed selectively in 28 symptomatic patients with normal endoscopy or LA grade A esophagitis. The mean DeMeester score and the number (percentage) of patients with pathological acid exposure are based on this subgroup (*n =* 28). For longitudinal comparison, the percentages (e.g., 17.0%) are expressed relative to the total cohort (*n =* 106). †Preoperative PPI use was for symptom control and not necessarily reflective of objective GERD diagnosis

Significant metabolic benefits were observed following LSG (Table 2). Fasting blood glucose and HbA1c levels showed marked and sustained improvement at 6 months and 1 year (*p <* 0.001 for both). Despite these systemic benefits, endoscopic findings revealed a progression of reflux pathology. The proportion of patients with severe erosive esophagitis (LA Grade C/D) increased from 1.9% preoperatively to 4.7% at 1 year. The persistence of Hill's Grade III/IV (61.3% at 1 year) and ZAP Grade ≥ II (32.1%) further indicates a refractory reflux phenotype, despite metabolic gains.

### Hill's classification as the primary predictor of GERD and its impact on disease-free survival

**Fig. 3:**
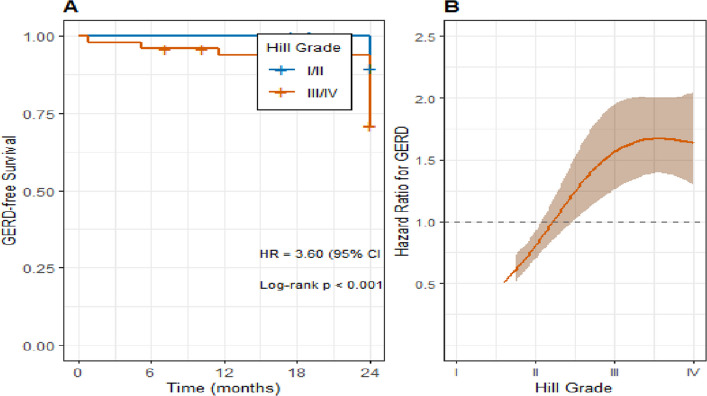
GERD-free survival after LSG according to preoperative Hill grade. **A** Kaplan–Meier curves for patients with Hill I/II (*n =* 44) vs Hill III/IV (*n =* 62). One-year GERD-free survival: Hill I/II = 71.4%, Hill III/IV = 41.9% (log-rank *p <* 0.001). **B** Restricted cubic spline showing the non-linear increase in hazard of GERD with increasing Hill grade (Cox model adjusted for age, sex, BMI, and %EWL). Statistical tests: Log-rank test and multivariable Cox proportional hazards model (HR and 95% CI shown in text)

Univariate analysis confirmed Hill's Grade III/IV, hiatal hernia, and LA Grade ≥ A as significant predictors of postoperative GERD (Table 3). In the multivariate model, which adjusted for these significant univariate predictors, only a preoperative Hill's grade of III or IV persisted as a strong and independent predictor (Adjusted OR: 3.92, 95% CI: 1.98—7.76, *p <* 0.001). The clinical impact of this finding is powerfully illustrated by time-to-event analysis. Kaplan–Meier curves (Fig. 3) showed a significant and early divergence in GERD-free survival between the risk groups (Log-rank test, *p <* 0.001). The GERD-free survival rate at 1 year was 71.4% for the Hill Grades I/II group, compared to only 41.9% for the Hill Grades III/IV group. Cox proportional hazards modeling confirmed that a high-risk Hill's grade increased the hazard of developing GERD at any time during the follow-up by a factor of 3.6 (HR: 3.60, 95% CI: 1.85—7.00, *p <* 0.001). Restricted cubic spline regression further revealed a non-linear increase in GERD risk with Hill's grade, with a steep rise beyond Grade II (*p <* 0.001), enhancing the predictive model’s granularity.

**Table 3:** Univariate and multivariate analysis of preoperative predictors for postoperative GERD

Predictor	Category	Univariate OR (95% CI)	*p*-value	Multivariate aOR (95% CI)	*p*-value
**Demographic Factors**					
Age	Per year increase	1.02 (0.98–1.06)	0.301	-	-
Sex	Female vs Male	1.25 (0.65–2.41)	0.502	-	-
Preoperative BMI	Per kg/m^2^	1.04 (0.97–1.11)	0.280	-	-
**Clinical & Endoscopic Factors**					
Hill's Classification	III/IV vs I/II	4.12 (2.15–7.89)	< 0.001	3.92 (1.98–7.76)	< 0.001
Hiatal Hernia	Present vs Absent	3.45 (1.58–7.52)	0.002	1.89 (0.78–4.59)	0.159
LA Classification	≥ A vs None	2.95 (1.45–6.00)	0.003	1.72 (0.78–3.82)	0.181
ZAP Classification	≥ II vs 0/I	1.89 (0.92–3.87)	0.083	-	-

*OR* Odds ratio, *aOR* adjusted odds ratio, *CI* Confidence interval, *BMI* Body mass index, *LA* Los Angeles, *ZAP* Z-line appearance; multivariate model adjusted for age, sex, BMI, and percentage excess weight loss (%EWL); Hosmer–Lemeshow *p =* 0.75, Nagelkerke R^2^ = 0.37. Regression analyses are based on pooled estimates from multiple imputation (m = 20)

To test the robustness of the primary association, we performed two sensitivity analyses. First, when patients with a preoperative hiatal hernia were excluded (*n =* 66 remaining; 106 total—40 with HH), Hill Grade III–IV remained independently associated with postoperative GERD (adjusted OR 3.62, 95% CI 1.74–7.50, *p <* 0.001). Second, restricting the definition of GERD to only those with pathological 24-h pH monitoring (DeMeester score > 14.7) produced a similarly strong association (adjusted OR 3.85, 95% CI 1.70–8.73). These consistent results across different analytical scenarios confirm the robustness of Hill’s classification as a predictor.

To evaluate the effect of surgical correction, we compared the postoperative incidence of GERD between patients without a hiatal hernia (*n =* 66) and those with a hiatal hernia who underwent concurrent posterior cruroplasty (*n =* 38). Postoperative GERD remained significantly more frequent in the cruroplasty group (52.6% [20/38] vs. 28.8% [19/66], *p =* 0.015), indicating that anatomical repair did not fully mitigate the reflux risk conferred by a defective gastroesophageal flap valve. This finding further supports the concept that intrinsic flap-valve competence, as reflected by Hill’s classification, is a stronger determinant of postoperative reflux than hiatal hernia presence alone. Table 3 includes preoperative predictors only; the impact of concurrent hiatal hernia repair is analyzed separately in the Results section.

### Sensitivity analysis using strict 2022 ACG-compliant GERD definition

“To ensure the robustness of our primary findings relative to contemporary diagnostic standards, we performed a sensitivity analysis using a stricter definition of GERD aligned with the 2022 ACG Clinical Guideline. In this analysis, GERD was redefined as LA grade B or higher esophagitis or pathological DeMeester score > 14.7, thereby excluding cases based solely on isolated LA grade A esophagitis (*n =* 8). Under this stricter definition, preoperative Hill Grades III–IV remained a strong and independent predictor of postoperative GERD (adjusted OR 3.71, 95% CI 1.82–7.55, *p <* 0.001). The results of the multivariate logistic regression under this definition are presented in Table 4.

**Table 4:** Multivariate analysis of preoperative predictors for postoperative GERD using a strict 2022 ACG-compliant definition*

Predictor	Category	Adjusted OR (95% CI)	*p*-value
Hill's Classification	III/IV vs I/II	**3.71 (1.82–7.55)**	**< 0.001**
Hiatal Hernia	Present vs Absent	1.85 (0.76–4.49)	0.173
LA Classification	≥ B vs None	1.65 (0.74–3.70)	0.221
Age (per year)	–	1.01 (0.97–1.05)	0.685
Sex (Female)	–	1.18 (0.60–2.31)	0.632
Preoperative BMI (per kg/m^2^)	–	1.03 (0.96–1.10)	0.398

^*^Model adjusted for age, sex, BMI, and %EWL. GERD is defined as LA grade B or higher esophagitis or a DeMeester score > 14.7. Hosmer–Lemeshow *p =* 0.82, Nagelkerke R^2^ = 0.36

### Stratification of esophagitis severity and its impact on quality of life

The preoperative Hill's grade effectively stratified patients' risk for the severity of postoperative esophageal injury. A strong positive correlation existed between the preoperative Hill's grade and the worst postoperative LA grade (Spearman's ρ = 0.59, *p <* 0.001). As detailed in Table 5, 88.9% of patients with a normal valve (Hill I) had no esophagitis at follow-up, whereas 44.4% of those with a patulous valve (Hill IV) developed moderate-to-severe esophagitis (LA Grade B-D). This progression had a direct and significant impact on patients' quality of life. At 1 year, patients with GERD reported significantly lower SF-36 scores compared to those without GERD, both physically (PCS: 42.1 vs. 47.3, *p =* 0.003) and mentally (MCS: 40.5 vs. 45.2, *p =* 0.009) (Table 6). Ordinal logistic regression confirmed a dose–response relationship, with an odds ratio of 2.10 (95% CI 1.65–2.68, *p <* 0.001) per unit increase in Hill's grade for escalating LA severity, reinforcing its prognostic utility.

**Table 5:** Correlation between preoperative Hill's grade and postoperative esophagitis severity

**Preoperative Hill's Grade**		**Postoperative LA Grade, n (%)**			
	**n**	**None**	**Grade A**	**Grade B**	**Grade C/D**
**Grade I**	18	16 (88.9%)	2 (11.1%)	0 (0%)	0 (0%)
**Grade II**	26	19 (73.1%)	5 (19.2%)	2 (7.7%)	0 (0%)
**Grade III**	35	20 (57.1%)	8 (22.9%)	5 (14.3%)	2 (5.7%)
**Grade IV**	27	13 (48.1%)	5 (18.5%)	6 (22.2%)	3 (11.1%)

*LA* Los Angeles, χ^2^ = 18.7, *p =* 0.002 (Chi-square test for association)

**Table 6:** Quality of life scores at 1 year by GERD status

Variable	No GERD (*n =* 67)	GERD (*n =* 39)	*p*-value
SF-36 Physical Component Score (PCS)	47.3 ± 5.9	42.1 ± 6.8	**0.003**
SF-36 Mental Component Score (MCS)	45.2 ± 6.3	40.5 ± 7.1	**0.009**

*PCS* Physical component score, *MCS* Mental component score, *p*-values from t-test comparing GERD vs. non-GERD groups

In addition to the cross-sectional association shown in Table 4, we further examined longitudinal changes in mucosal findings to assess within-patient progression. To evaluate the structural evolution of mucosal injury over time, we compared preoperative and postoperative endoscopic findings. A moderate positive correlation was observed between preoperative and postoperative Los Angeles (LA) grades (Spearman’s ρ = 0.48, *p <* 0.001). Among patients without preoperative esophagitis (*n =* 73), 79.5% (*n =* 58) remained free of esophagitis postoperatively, whereas 15.1% (*n =* 11) developed LA Grade A and 5.5% (*n =* 4) progressed to LA Grade B. Similarly, preoperative Z-line appearance (ZAP) grade correlated significantly with postoperative ZAP grade (ρ = 0.51, *p <* 0.001). Of those with a normal or near-normal Z-line (ZAP 0/I) at baseline (*n =* 75), 85.3% (*n =* 64) maintained this status, while 14.7% (*n =* 11) developed ZAP ≥ II changes.

These findings indicate that baseline mucosal integrity partially predicts postoperative mucosal deterioration, further validating the prognostic role of preoperative endoscopic grading in GERD development after LSG.

### Machine learning model outperforms traditional regression for GERD prediction

**Fig. 4:**
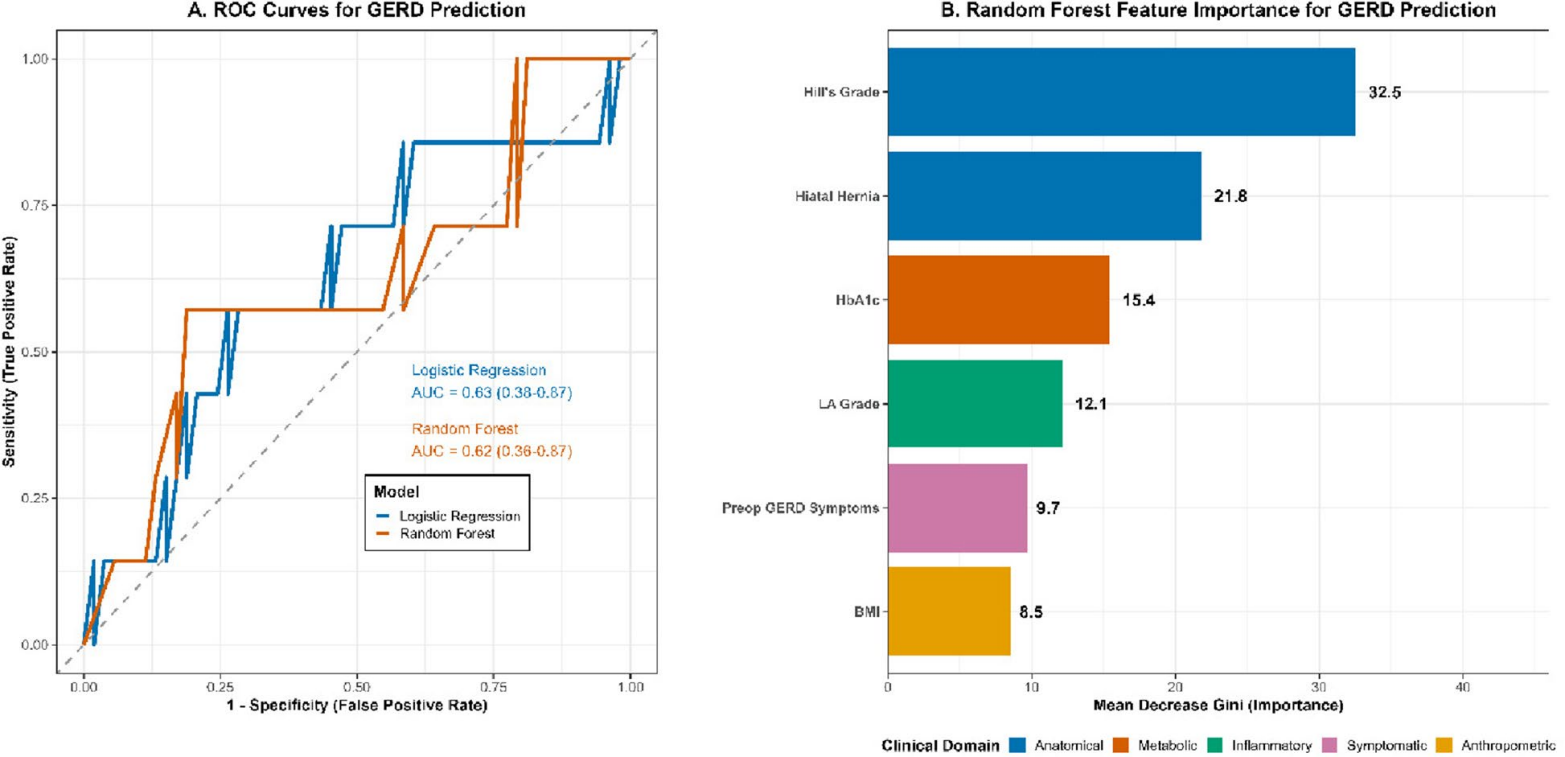
ROC curves and feature-importance ranking for Random Forest versus multivariate logistic regression predicting 1-year postoperative GERD. **A** ROC comparison between the Random Forest (RF) model and the multivariate logistic regression model. AUC_RF = 0.84 (95% CI 0.76–0.91); AUC_logistic = 0.76 (95% CI 0.67–0.85); DeLong’s test for comparison, *p =* 0.03. **B** Feature-importance ranking from the RF model based on Mean Decrease in Gini. Hill's grade was the most influential predictor. Modeling details: The RF model was trained on a 70% training/30% test split with fivefold cross-validation; see Methods for details

We evaluated whether a machine learning (ML) approach could provide superior predictive accuracy for post-LSG GERD compared to traditional logistic regression and sought to identify the most important predictive features. While traditional regression confirmed Hill's grade as a key predictor, we developed a Random Forest classifier to capture potential complex, non-linear interactions. The ML model demonstrated superior performance, achieving an area under the receiver operating characteristic curve (AUC) of 0.84 (95% CI: 0.76–0.91) compared to 0.76 (95% CI: 0.67–0.85) for the multivariate logistic regression model (*p =* 0.03 for DeLong's test). Feature importance analysis (Table 7) confirmed Preoperative Hill's Grade as the dominant predictor, but also identified Hiatal Hernia and HbA1c as key contributors, suggesting a synergistic role of anatomical and metabolic factors (Fig. 4). Calibration analysis showed excellent agreement between predicted and observed GERD probabilities (Brier score = 0.09). Calibration plots demonstrated minimal deviation from the 45° reference line. Although the model achieved strong internal discrimination (AUC = 0.84), external validation in an independent cohort will be required to confirm generalizability and clinical utility.

**Table 7:** Machine Learning Feature Importance for Predicting Postoperative GERD

Predictor	Mean Decrease Gini	Clinical Domain
Hill's Grade	32.5	Anatomical
Hiatal Hernia	21.8	Anatomical
HbA1c	15.4	Metabolic
LA Grade	12.1	Inflammatory
Preop GERD Symptoms	9.7	Symptomatic
BMI	8.5	Anthropometric

The "Mean Decrease Gini" represents the feature's importance in the Random Forest model; a higher value indicates greater predictive power

### Identification of distinct GERD phenotypes viatrajectory analysis

**Fig. 5:**
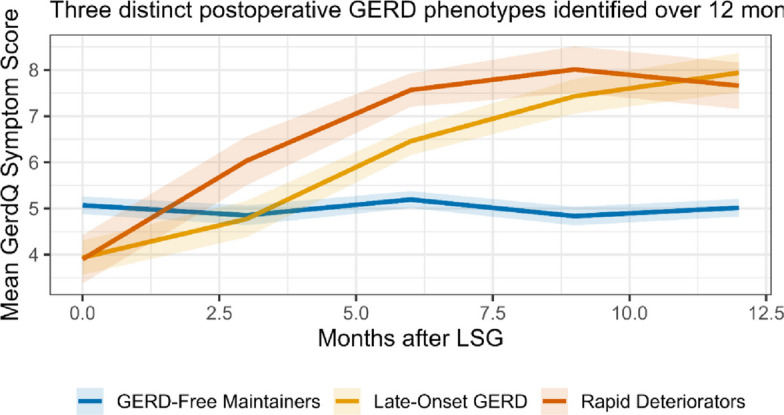
Latent class mixed model (LCMM) analysis identifying distinct trajectories of GERD symptoms after LSG. GerdQ scores were modeled over 12 months postoperatively, revealing three distinct phenotypes: 'GERD-Free Maintainers' (*n =* 62, 58.5%) with persistently low scores; 'Late-Onset GERD' (*n =* 25, 23.6%) with significant symptom development after 6 months; and 'Rapid Deteriorators' (*n =* 19, 17.9%) with an early, steep rise in symptoms within 3 months. The three-class solution was selected based on the optimal Bayesian Information Criterion (BIC). Baseline profiling (Table 8) identified the 'Rapid Deteriorator' phenotype as being overwhelmingly characterized by preoperative Hill’s Grade IV

To move beyond a binary GERD outcome, we identified distinct patterns, or phenotypes, of GERD development over time. Latent Class Mixed Model analysis of longitudinal GerdQ scores revealed three distinct trajectories of GERD symptom severity over the 12-month follow-up (Fig. 5). The model-fit indices (Bayesian Information Criterion) strongly supported this three-class solution. The largest group was “GERD-Free Maintainers” (58.5%), who had minimal symptoms throughout. The “Late-Onset GERD” group (23.6%) developed significant symptoms after the 6-month mark, coinciding with solid-diet normalization. Most critically, a “Rapid Deteriorator” phenotype (17.9%) exhibited a sharp increase in symptoms within the first 3 months. Analysis of the baseline characteristics of these phenotypes revealed that the Rapid Deteriorator group was overwhelmingly characterized by a preoperative Hill’s Grade IV (89.5% vs. 4.8% in GERD-Free Maintainers, *p <* 0.001) and had a higher prevalence of hiatal hernia, providing a clear clinical profile for the highest-risk patients (Table 8).

**Table 8:** Baseline Characteristics of the Identified GERD Phenotypes

Characteristic	GERD-Free Maintainers (*n =* 62)	Late-Onset GERD (*n =* 25)	Rapid Deteriorators (*n =* 19)	Overall *p-*value	Pairwise Comparisons *(p-*value*)*
**Preoperative Endoscopic Findings**					
Hill's Grade IV, n (%)	3 (4.8%)	7 (28.0%)	17 (89.5%)	< 0.001	GF vs. LO: 0.003; GF vs. RD: < 0.001; LO vs. RD: < 0.001
Hiatal Hernia, n (%)	18 (29.0%)	11 (44.0%)	11 (57.9%)	0.045	GF vs. LO: 0.185 GF vs. RD: 0.021 LO vs. RD: 0.350
LA Grade ≥ A, n (%)	16 (25.8%)	9 (36.0%)	8 (42.1%)	0.281	-
**Clinical & Metabolic Factors**					
Type 2 Diabetes, n (%)	22 (35.5%)	11 (44.0%)	10 (52.6%)	0.341	-
Preop BMI (kg/m^2^), mean ± SD	45.8 ± 5.5	46.5 ± 6.1	47.8 ± 6.5	0.387	-

*GF* GERD-Free Maintainers, *LO* Late-Onset GERD, *RD* Rapid Deteriorators, *LA* Los Angeles, *BMI* Body Mass Index. Overall p-values from the Chi-square test or ANOVA. Pairwise p-values are reported for characteristics with a significant overall test (*p <* 0.05) and were derived from post-hoc tests with Bonferroni adjustment (categorical) or Tukey’s HSD (continuous)

### Interaction analysis reveals synergy between anatomical and metabolic risk factors

We investigated whether the presence of type 2 diabetes mellitus (T2DM) amplifies the GERD risk associated with a defective gastroesophageal flap valve. A multiplicative interaction term was added to the multivariate model to test this hypothesis. The analysis revealed a significant positive interaction between Hill's Grade (III/IV) and T2DM (Adjusted OR for interaction: 2.45, 95% CI: 1.18–5.08, *p =* 0.016). This indicates that the risk of GERD in patients with *both* a high Hill's grade and T2DM is greater than the sum of their individual risks. As shown in Table 8, the predicted probability of GERD was highest (68.4%) in this subgroup, suggesting a synergistic pathophysiological mechanism involving both mechanical incompetence and diabetic neuropathy/motility disorders (Table 9).

**Table 9:** Stratified analysis: predicted probability of GERD by Hill's grade and diabetic status

Preoperative Hill's Grade	No Diabetes (*n =* 63)	Type 2 Diabetes (*n =* 43)	p-value for interaction
Grade I/II	18.2%	22.7%	**0.016**
Grade III/IV	41.5%	68.4%	

Probabilities are adjusted for age, sex, BMI, and hiatal hernia. The significant *p*-value for interaction confirms the effect of Hill's grade on GERD risk is modified by diabetic status

### The differential impact of GERD phenotypes on quality of life

To assess whether the newly identified GERD phenotypes have a differential impact on patients' quality of life, beyond the binary presence or absence of GERD. When QoL outcomes were analyzed according to the GERD phenotypes, a clear gradient was observed (Table 10). The "Rapid Deteriorator" group reported the worst outcomes, with Physical Component Scores (PCS) significantly lower than both the "Late-Onset" group and the GERD-Free Maintainers (*p <* 0.001). This demonstrates that the *pattern* of GERD development is a critical determinant of patient-centered outcomes, with early, aggressive symptom onset having the most profound negative impact on the perceived success of surgery.

**Table 10:** Quality of life at 1 year stratified by GERD phenotype

GERD Phenotype	n	SF-36 PCS (Mean ± SD)	SF-36 MCS (Mean ± SD)
**GERD-Free Maintainers**	62	48.1 ± 5.7	46.0 ± 6.1
**Late-Onset GERD**	25	43.5 ± 6.1	41.8 ± 6.9
**Rapid Deteriorators**	19	38.9 ± 6.5	37.2 ± 7.3
**p-value (ANOVA)**		< 0.001	< 0.001
**Post-hoc Comparisons (Tukey test)**			
Maintainers vs. Late-Onset		0.003	0.009
Maintainers vs. Deteriorators		< 0.001	< 0.001
Late-Onset vs. Deteriorators		0.021	0.033

*PCS* Physical Component Score, *MCS* Mental Component Score, *SD* Standard Deviation. *p*-values indicate statistical significance (*p <* 0.05)

## Discussion

The global obesity epidemic continues to drive the prevalence of numerous chronic conditions, with gastroesophageal reflux disease (GERD) standing as a prominent comorbidity [1, 2]. In Egypt, where more than one-third of adults are obese [3], bariatric surgery, particularly laparoscopic sleeve gastrectomy (LSG), is increasingly recognized as an effective long-term solution for morbid obesity. However, the impact of LSG on the development or course of postoperative reflux remains a point of significant clinical controversy.

This prospective multicenter study evaluated whether preoperative endoscopic assessment of the gastroesophageal junction, specifically Hill’s classification, predicts the development of GERD after LSG. We found that preoperative Hill Grades III–IV were strongly and independently associated with the development of objective GERD at 6 months and 1 year **(**adjusted OR = 3.92, 95% CI 1.98–7.76, *p <* 0.001; HR = 3.60, 95% CI 1.85–7.00, *p <* 0.001). Higher Hill grades also correlated with greater esophagitis severity and poorer health-related quality of life (Tables 1, 2, 3, 4, 5 and 6, Fig. 3). These associations persisted after adjustment for age, sex, BMI, % EWL, and other endoscopic parameters, and were reinforced by machine-learning and trajectory analyses that identified a high-risk “Rapid Deteriorator” phenotype overwhelmingly concentrated among patients with Hill Grade IV (Tables 3, 7, 8, 9 and 10; Figs. 4 and 5). Together, our findings indicate that routine preoperative endoscopic evaluation incorporating Hill’s grade provides clinically actionable prognostic information for Egyptian patients undergoing primary LSG [15–17].

It is important to emphasize that GERD in this study was diagnosed using strict objective criteria combining endoscopic and pH-metric data, not symptom scores alone. This approach minimizes misclassification and aligns with contemporary GERD guidelines. Our pH-metric results (Table 2) confirmed that 41.5% of patients had pathological acid exposure preoperatively, and postoperatively, 64.3% of tested symptomatic patients had confirmed pathological reflux, contributing to 11.3% of GERD diagnoses based on pH criteria alone. Despite a substantial baseline burden of reflux symptoms (mean GerdQ 7.2 ± 3.1; Table 1), symptom scores alone showed limited predictive value, as demonstrated by the non-significant difference in preoperative GerdQ scores between those who later developed GERD and those who did not (*p =* 0.089).

We recognize the 2022 ACG Clinical Guideline, which advises that LA grade A esophagitis alone is insufficient for GERD diagnosis without abnormal reflux monitoring. To ensure the robustness of our primary finding against this contemporary standard, we performed a sensitivity analysis using a stricter, ACG-aligned GERD definition (LA Grade B or higher, or pathological pH monitoring). As shown in Table 4, the association between preoperative Hill Grade III–IV and postoperative GERD remained strong and statistically significant (adjusted OR 3.71, 95% CI 1.82–7.55, *p <* 0.001). This confirms that the predictive power of Hill’s classification is not dependent on the inclusion of isolated LA Grade A findings and is valid under current diagnostic guidelines, reinforcing Hill grade as a fundamental, guideline-independent predictor of reflux risk after LSG.

Our analyses revealed that patients with preoperative Hill Grade III or IV were at substantially higher risk of developing GERD after LSG. Even after accounting for confounders such as age, sex, BMI, %EWL, and other endoscopic findings, Hill Grade III-IV remained the single strongest independent predictor (multivariate OR = 3.92 [95% CI 1.98–7.76], *p <* 0.001). Time-to-event (survival) curves demonstrate substantially reduced GERD-free survival in those high-grade Hill groups (Fig. 3, Table 3). Additionally, there was a monotonic (dose–response) relationship: as the preoperative Hill grade increases, postoperative LA esophagitis grade worsens (Spearman’s ρ = 0.59) (Table 5).

The longitudinal correlation between preoperative and postoperative endoscopic findings reinforces the structural basis of reflux progression after LSG. We observed a moderate positive correlation between preoperative and postoperative LA grades (Spearman’s ρ = 0.48, *p <* 0.001) and between Z-line appearance (ZAP) grades (ρ = 0.51, *p <* 0.001). Most patients with intact mucosa at baseline remained disease-free postoperatively, whereas those with mild preoperative abnormalities were more likely to progress to erosive changes. These data suggest that preexisting microscopic or subclinical mucosal vulnerability persists after LSG and contributes to the observed variability in postoperative reflux trajectories. Together with the cross-sectional association demonstrated in Table 5, this longitudinal finding underscores that mucosal integrity and flap-valve competence act synergistically in determining postoperative reflux risk.

The clinical burden of postoperative GERD was evident in our cohort: nearly one-third of patients (29.2%) required daily proton-pump inhibitor therapy at one year, and 2.8% underwent conversion to Roux-en-Y gastric bypass for severe, refractory symptoms (Table 2). These intervention rates highlight that postoperative reflux is not only an endoscopic or pH-metric diagnosis but a condition with meaningful therapeutic and surgical consequences. This reinforces the importance of preoperative anatomical assessment (Hill’s grade) to identify patients at risk of clinically significant GERD requiring long-term medical or even revisional surgical management.

Notably, two patients in our cohort presented with preoperative LA Grade C/D esophagitis and underwent LSG after failure of optimized medical therapy and detailed informed consent regarding reflux risk, prior to the widespread adoption of the 2020 International Consensus recommendations. Both developed persistent postoperative GERD, reinforcing the guideline that severe erosive esophagitis should favor RYGB over LSG. This real-world experience underscores the importance of integrating contemporary reflux guidelines into preoperative decision-making, particularly in patients with high-risk endoscopic phenotypes.

Our findings are consistent with and extend prior evidence linking gastroesophageal flap valve (GEFV) morphology to postoperative reflux outcomes. Chue et al*.* [16] prospectively evaluated 241 patients undergoing LSG and demonstrated that higher preoperative Hill grades were significantly associated with postoperative GERD and erosive esophagitis at one year. In their multivariate model, Hill Grade III conferred an odds ratio (OR) of 19.4 (95% CI 7.3–51.4; *p <* 0.001) for postoperative GERD compared with Hill Grade I, while Hill Grade IV carried an OR of 24.6 (95% CI 8.8–68.9; *p <* 0.001). Similarly, Alvarez et al. [17]. analyzed 360 consecutive LSG patients and reported that Hill Grades III–IV independently predicted postoperative reflux (adjusted OR = 1.94, 95% CI 1.11–3.37; *p =* 0.019) even after controlling for baseline symptoms, hiatal hernia, and BMI. Importantly, concurrent hiatal hernia repair did not eliminate the elevated risk, underscoring the intrinsic prognostic importance of the GEFV configuration itself. Our own data corroborate this observation. Despite standardized posterior cruroplasty being performed in all patients with hiatal hernia, postoperative GERD remained significantly more frequent in this subgroup (52.6% vs. 28.8%, *p =* 0.015). This suggests that anatomical repair alone may not fully restore flap-valve competence once the gastroesophageal junction architecture is compromised. Even after adequate hiatal closure, the functional integrity of the valve, as captured by Hill’s classification, appears to remain the dominant determinant of reflux risk. These findings highlight the limited protective effect of concurrent cruroplasty in patients with advanced GEFV deterioration and further emphasize the need for individualized surgical decision-making based on preoperative Hill grading.

Unlike studies limited to symptom reporting or esophagitis prevalence, our analysis integrates endoscopic grading, objective pH-metric data, and longitudinal trajectory modeling, showing that the Hill grade not only predicts whether GERD develops but also influences its onset and progression speed, exemplified by the “Rapid Deteriorator” phenotype concentrated in patients with Hill Grade IV. The inclusion of pH monitoring allowed us to identify non-erosive GERD cases, reinforcing that anatomical risk (Hill grade) predicts both erosive and non-erosive reflux phenotypes.

Mechanistically, our results highlight the pivotal role of the gastroesophageal flap valve (GEFV) in maintaining the anti-reflux barrier. Higher Hill grades denote structural weakness of this valve, compromising its closure and reducing the efficiency of pressure transmission to the lower esophageal sphincter (LES). The LSG procedure introduces further mechanical challenges; resection of the gastric fundus and creation of a tubular gastric conduit are known to increase intragastric pressure and alter the geometry of the esophagogastric junction, thereby creating an environment more susceptible to reflux [18–20]. These combined factors explain the magnitude of the observed association. The significant dose–response relationship (Spearman ρ = 0.59, *p <* 0.001) further demonstrates that as Hill grade worsens, reflux severity increases quantitatively. This underscores the practical importance of including Hill grading in preoperative EGD reports to guide risk assessment and surgical decision-making.

Although patients exhibited significant metabolic and glycemic improvements post-LSG (Table 2), the incidence of objective GERD at one year reached 36.8%, and the prevalence of severe esophagitis (LA grade C or D) rose modestly (Table 2). Moreover, those who developed GERD showed significantly lower SF-36 physical and mental scores at one year compared to those without GERD, indicating worse patient-reported quality of life (Table 6).

These observations align with multiple systematic reviews and meta-analyses demonstrating that LSG is frequently associated with de novo or worsened reflux and esophagitis compared with Roux-en-Y gastric bypass (RYGB). For example, Yeung et al. reported a pooled postoperative GERD prevalence of 19–23% and esophagitis in 30% of patients, while Barrett’s esophagus developed in 6% after LSG [8]. Similar findings were confirmed by Oor et *al*. and Gu et *al*., both showing significantly higher reflux rates and PPI dependence after sleeve than bypass procedures [19, 21].

While our findings align with a growing body of objective evidence linking LSG to increased GERD risk, it is important to acknowledge that some reports have described stable or even improved reflux symptoms postoperatively [12, 13]. These divergent outcomes likely stem from methodological variations across studies. Notably, many reports of favorable reflux outcomes rely primarily on symptom questionnaires rather than objective endoscopic or pH-metric confirmation [12]. Furthermore, inconsistencies in surgical technique, including variable rates of concurrent hiatal hernia repair, differences in bougie size, and stapling distance from the esophagus, may significantly influence postoperative reflux dynamics [13, 20]. Such heterogeneity, combined with differences in GERD definitions and follow-up duration, underscores the need for standardized, objective outcome assessment, as employed in the present study.

Clinically, these data emphasize that metabolic benefit does not necessarily translate to gastroesophageal protection. In anatomically susceptible patients, particularly those with pre-existing GEFV incompetence or high Hill grades, the geometric and pressure changes induced by the sleeve can unmask or exacerbate reflux-related mucosal injury.

In our cohort, this was evidenced by the significant increase in erosive esophagitis. It is a critical finding, however, that systematic histopathological analysis at one year did not detect any progression to intestinal metaplasia (Barrett’s esophagus), despite the observed mechanical and inflammatory insults. This suggests that the one-year postoperative period may precede the threshold for metaplastic transformation, underscoring the importance of this window for aggressive medical management and endoscopic surveillance to prevent long-term sequelae.

From a physiological standpoint, sleeve gastrectomy reshapes the stomach into a narrow, less compliant reservoir that elevates intragastric pressure and modifies the gastroesophageal junction anatomy, reducing the LES’s competence to prevent reflux [19, 21]. These structural and pressure-related changes account for the persistence or worsening of GERD despite metabolic improvement. The significant association between postoperative GERD and lower SF-36 scores confirms that reflux meaningfully impairs quality of life. Longitudinal studies have similarly shown that persistent reflux reduces both physical and mental well-being, partially offsetting the expected benefits of weight loss [22].

Taken together, these results reinforce that metabolic success does not equate to esophageal safety. This duality, systemic improvement but local risk, must be discussed with patients pre-operatively and integrated into postoperative surveillance protocols.

We trained a Random Forest (RF) classifier, which demonstrated improved discrimination in internal testing compared to conventional logistic regression (AUC = 0.84 vs 0.76). This level of discrimination (AUC > 0.80) is generally regarded as excellent for clinical prediction models and is comparable to the performance of other recently developed AI tools in bariatric surgery [23]. However, these results should be interpreted as exploratory, as the model was trained and tested internally; external validation and prospective evaluation are necessary before clinical deployment. In the RF model, Hill's grade emerged as the most important predictor, followed by hiatal hernia presence and preoperative HbA1c (Table 7, Fig. 4). Using latent class trajectory modeling, we delineated three clinically meaningful phenotypes of postoperative GERD risk: GERD-Free Maintainers, Late-Onset, and Rapid Deteriorators. The Rapid Deteriorator group was overwhelmingly enriched for patients with preoperative Hill Grade IV (Table 8, Fig. 5).

The superiority of machine learning (ML) approaches, particularly ensemble methods like RF, in capturing complex, nonlinear interactions among predictors is increasingly recognized in bariatric outcome studies. For example, Emile et al. built an AI model to predict de novo GERD after sleeve gastrectomy using a cohort of 441 patients; their model achieved an AUC of 0.93 (95% CI 0.88–0.99), with top predictive features including age, baseline GERD status, and technical surgical parameters (bougie size, stapler distance) [23]. Their approach confirms that integrating multiple risk dimensions, anatomical, metabolic, and procedural, can meaningfully improve predictive performance. However, their model did not incorporate standardized endoscopic grading (e.g., Hill classification), which limits translation.

Our work advances beyond that because we explicitly integrate Hill’s classification into the predictive pipeline and further refine risk stratification through phenotype modeling. The identification of a Rapid Deteriorator phenotype offers prognostic granularity not captured in prior studies: it distinguishes which high-risk patients, particularly those with Hill Grade IV, develop early and clinically significant GERD from those with slower or milder trajectories. This finding has direct clinical implications, as it supports early, phenotype-guided surveillance and prophylactic strategies, such as intensified acid suppression or revisional consideration in anatomically vulnerable patients.

Mechanistically, the Random Forest model’s prioritization of Hill’s grade and hiatal hernia underscores that structural anatomy remains the foundation upon which metabolic and surgical factors act. In contrast, HbA1c emerged as a key metabolic modifier, suggesting that chronic hyperglycemia may exacerbate reflux via autonomic neuropathy, impaired gastric emptying, and altered mucosal repair capacity, as previously reported in diabetic reflux physiology studies [24, 25]. The phenotype-based approach further implies that patients with pre-existing GEFV compromise (Hill IV) may exhibit a threshold effect: once surgical stress alters pressure dynamics and geometry, they rapidly deteriorate in terms of acid exposure, mucosal injury, and symptomatic burden.

Such integration of objective endoscopic grading with data-driven phenotyping aligns with emerging applications of machine learning in bariatric surgery, where ML approaches have shown potential for enhanced predictive modeling compared to traditional regression methods [23–25]. Ultimately, combining explainable ML with validated anatomical indices like Hill’s grade provides a balanced, interpretable, and clinically actionable framework for individualized postoperative risk stratification.

Our trajectory classification also aligns with the evolving paradigm in precision surgery: not all postoperative reflux is identical, and early interventions (medical, endoscopic, or revisional) may be most beneficial in a defined “high-decline” subgroup. Future models might incorporate time-varying covariates (e.g., weight regain, diet compliance) to further refine these phenotypes.

Our interaction analysis revealed that type 2 diabetes mellitus (T2DM) significantly augments the reflux risk associated with an incompetent gastroesophageal flap valve. In patients with Hill Grade III/IV and concurrent diabetes, the predicted probability of postoperative GERD rose to 68%, substantially higher than in non-diabetic counterparts (Table 9).

This interaction likely reflects the combined influence of two synergistic factors: structural deficiency of the gastroesophageal valve and diabetes-related functional disturbances of the upper gastrointestinal tract. When the flap valve is compromised, rising intragastric pressure favors reflux through the weakened junction. Diabetes intensifies this tendency through autonomic neuropathy–induced motility impairment, reduced esophageal clearance, and diminished buffering capacity, which collectively prolong mucosal acid exposure [26–28]. Additionally, diabetic gastroparesis elevates intragastric pressure and delays emptying, further predisposing to reflux in the presence of an anatomically weak barrier [26–28].

Beyond these motility disturbances, chronic hyperglycemia has been shown to impair mucosal defense and repair through microvascular dysfunction, oxidative stress, and collagen glycation, thereby reducing tissue resilience to acid-related injury [29, 30]. The elevated HbA1c values in our diabetic cohort likely mirror this pathological milieu, which amplifies mechanical susceptibility to reflux. Consequently, the combination of a structurally deficient valve and a metabolically impaired host produces a marked escalation in both the frequency and severity of GERD manifestations.

These interpretations align with prior reports emphasizing the complex interplay between mechanical and functional factors in post-sleeve reflux. Johari et *al*. [26] described the multifactorial origins of reflux after LSG, highlighting that impaired gastric compliance, high intragastric pressure, and delayed emptying act synergistically to increase acid exposure. Another study [27] further demonstrated that abnormal preoperative pH parameters, including elevated DeMeester scores, predict postoperative reflux burden, underscoring the need for integrated anatomical and physiological assessment. In addition, studies of GERD in diabetic populations confirm that esophageal clearance is slower and acid exposure time longer than in non-diabetic controls, even in the absence of structural lesions [28].

Taken together, these findings reinforce that anatomical and metabolic factors act in concert rather than in isolation. Diabetes magnifies the pathological consequences of an incompetent flap valve, producing an exponential, not additive, increase in reflux propensity. Clinically, this insight supports incorporating metabolic status into preoperative endoscopic risk models and considering functional testing (pH or manometry) when both diabetes and high Hill grade coexist. Tailored surgical decision-making and intensified postoperative surveillance may therefore mitigate reflux-related morbidity in this high-risk subgroup.

Our phenotype-based analysis demonstrated that the "Rapid Deteriorator" group not only experienced earlier and more severe GERD events (Fig. 5) but also exhibited the lowest SF-36 physical and mental health scores at follow-up compared with the other phenotypes (Table 10). This finding confirms that postoperative reflux trajectories directly translate into patient-centered outcomes; individuals in the high-risk phenotype sustain a disproportionate loss of quality of life despite comparable metabolic benefits.

This observation is consistent with long-term bariatric literature showing that reflux substantially undermines postoperative well-being. Felsenreich et al. found that patients with symptomatic reflux ten years after sleeve gastrectomy reported significantly lower SF-36 scores across all domains, including physical functioning and general health perception [31]. Okuyan et al. similarly observed that GERD symptoms and suboptimal %EWL after sleeve were jointly associated with poorer health-related quality of life, particularly in physical and role-limitation dimensions [32]. Lagergren et al. further confirmed that chronic reflux is independently linked to reduced vitality and social functioning even outside bariatric cohorts [33].

Mechanistically, the association between phenotype and QoL likely reflects sustained mucosal inflammation, persistent chest discomfort, and dietary restriction, which collectively diminish both physical capacity and psychological satisfaction. Moreover, patients with recurrent or severe reflux often experience sleep disturbance, chronic cough, and anxiety regarding symptom recurrence, compounding the subjective burden [33, 34].

From a clinical standpoint, phenotype stratification offers a pragmatic framework for precision follow-up. Recognizing membership in the Rapid Deteriorator group should prompt early diagnostic endoscopy, tighter acid suppression, and consideration of revisional procedures before irreversible mucosal damage develops. Integrating phenotypic information into counseling helps set realistic expectations and directs preventive interventions to those at highest risk. In essence, phenotype modeling bridges the gap between predictive analytics and tangible patient outcomes: it quantifies not only who develops reflux but also how profoundly it affects their quality of life, adding critical translational value to postoperative care.

Taken together, our data converge on a unified mechanistic and clinical narrative: structural incompetence of the gastroesophageal junction, as quantified by Hill Grades III–IV, emerges as the dominant and reproducible predictor of post-LSG GERD in this multicenter Egyptian cohort. The presence of a compromised flap valve dictates the trajectory of reflux despite the substantial metabolic and glycemic improvements conferred by LSG. Machine learning and trajectory modeling deepen this understanding by demonstrating that Hill-based anatomical vulnerability translates into distinct patient phenotypes and by improving predictive discrimination beyond traditional regression frameworks. These results resonate with recent multicenter and meta-analytic evidence showing that mechanical determinants, rather than metabolic outcomes, largely govern postoperative reflux risk, and that Roux-en-Y gastric bypass (RYGB) consistently mitigates long-term reflux compared with sleeve gastrectomy [35–37].

This study advances the field both globally and regionally. Internationally, it introduces a methodological synthesis that combines prospective, multicenter data with rigorous standardized endoscopic grading (Hill, LA, ZAP) and modern predictive analytics (Random Forest, LCMM). Unlike prior single-center or retrospective studies, we applied longitudinal objective endpoints at both six months and one year to capture dynamic reflux trajectories. Regionally, this is the first Egyptian prospective cohort to systematically integrate endoscopic risk stratification, machine learning, and phenotype modeling. This fills a crucial evidence gap within the MENA region, where sleeve gastrectomy is increasingly dominant, but population-specific data on post-LSG GERD remain scarce [38, 39].

Although all participating centers were located within Egypt, regional factors such as dietary composition, obesity phenotype, and referral patterns may influence baseline GERD prevalence. However, Hill’s classification reflects anatomical competence of the gastroesophageal flap valve, a mechanism shown to predict reflux across diverse populations. Therefore, while absolute GERD rates may vary, the predictive value of Hill grading is likely generalizable beyond this regional context. External validation in non-MENA cohorts remains warranted.

From an implementation perspective, Hill grading can be readily incorporated into routine preoperative endoscopy without additional equipment or cost. The high inter-observer agreement observed in this study supports its reproducibility in real-world practice, even in resource-limited settings, provided that basic endoscopic training and standardized image acquisition are applied. From a clinical standpoint, our findings strongly support routine preoperative esophagogastroduodenoscopy (EGD) with explicit documentation of Hill’s grade as part of the bariatric evaluation. Patients with Hill Grade III–IV, particularly those with coexisting type 2 diabetes or baseline esophagitis, represent a high-risk subgroup who should receive tailored counseling about reflux risk and may be better suited for RYGB when anti-reflux benefit outweighs restrictive advantages. Moreover, early postoperative surveillance (EGD at 6–12 months, low-threshold pH testing) should be prioritized in these individuals.

The demonstrated superiority of our machine-learning models indicates an imminent role for integrated predictive platforms that combine anatomical (Hill grade, hiatal hernia), metabolic (HbA1c, BMI), and technical variables to refine procedure selection and informed consent. Such precision frameworks are aligned with contemporary bariatric paradigms that emphasize individualized risk modeling rather than one-size-fits-all surgical choices [40, 41]. Importantly, these implications are corroborated by recent meta-analyses, which confirm that LRYGB confers significantly lower long-term reflux and revisional surgery rates than LSG, further validating our conclusions [37, 42].

In summary, this prospective, multicenter Egyptian cohort demonstrates that preoperative Hill Grade III–IV is a reproducible and independent predictor of objective GERD after LSG, with implications for both patient counseling and procedure selection. Hill grade improves risk stratification when combined with anatomical (hiatal hernia) and metabolic (HbA1c) variables, and identifies a high-risk “Rapid Deteriorator” phenotype that may benefit from intensified early surveillance or consideration of alternative bariatric procedures. These findings support routine documentation of Hill grade during preoperative EGD and motivate prospective trials of Hill-guided surgical selection.

Several limitations merit acknowledgment. First, although the one-year follow-up was sufficient to characterize early and mid-term GERD and esophagitis, longer surveillance is essential to assess late outcomes such as Barrett’s esophagus and neoplastic progression. Second, while multicenter, the cohort represents a regional Egyptian population; external validation in other healthcare contexts will enhance generalizability. Third, although objective testing (endoscopy ± selective 24-h pH monitoring) was used, universal pre- and postoperative impedance/pH-manometry for all patients was not feasible due to resource constraints—a limitation common to large, real-world cohorts. Consequently, quantification of acid and non-acid exposure is limited to the subset who underwent pH testing, and some asymptomatic GERD cases may have been missed. In addition, the absence of routine pH-impedance monitoring may have resulted in underestimation of non-acid or weakly acidic reflux, particularly in symptomatic patients with normal endoscopic findings. Nevertheless, pH monitoring was systematically applied per protocol and contributed meaningfully to the diagnosis of objective GERD in 11.3% of cases. Fourth, although the Random Forest and latent class trajectory models achieved robust internal validity, external calibration and cross-institutional validation remain prerequisites for clinical deployment. Finally, while we adjusted for major confounders (age, sex, BMI, %EWL), residual confounding and center-specific variations in surgical technique may still influence outcomes.Although a standardized laparoscopic sleeve gastrectomy protocol was followed across participating centers—including uniform bougie size, sleeve orientation, and staple line construction—minor variations in operative details such as the exact distance from the pylorus and technical nuances of crural repair may have occurred. These differences, inherent to multicenter surgical practice, could have contributed to variability in postoperative reflux outcomes.

Future research should build on these findings through extended longitudinal surveillance, ideally spanning five years or more, to delineate the true natural history of reflux-related sequelae such as Barrett’s esophagus and early neoplasia after LSG. Equally important are comparative or randomized clinical trials evaluating procedure selection strategies guided by preoperative Hill grading, which could definitively determine whether individualized surgical planning mitigates reflux risk without compromising metabolic benefit. Moreover, prospective external validation of the predictive algorithms developed in this work, incorporating assessments of cost-effectiveness and implementation feasibility in resource-limited health systems, will be crucial for translating these precision models into real-world practice.

## Conclusion

In this prospective multicenter Egyptian cohort, preoperative Hill’s classification of the gastroesophageal flap valve proved to be a powerful, independent predictor of GERD following laparoscopic sleeve gastrectomy, accurately stratifying risk for clinically significant esophagitis and impaired quality of life. Incorporating Hill’s grading into routine preoperative endoscopic evaluation and predictive algorithms, including machine-learning models, may substantially enhance patient counseling, individualized surgical selection, and tailored postoperative surveillance. Patients with Hill Grade III–IV, particularly those with concomitant type 2 diabetes mellitus, represent a high-risk subgroup in whom the reflux-protective advantage of Roux-en-Y gastric bypass should be carefully weighed against metabolic and procedural factors during shared decision-making. These findings support standardized adoption of preoperative endoscopic grading in bariatric programs and provide a framework for future longitudinal and interventional research to optimize reflux outcomes both globally and within Egypt’s expanding bariatric population.

## Data Availability

All relevant data are included in this published article.

## Abbreviations

ACG: American College of Gastroenterology
ALT: Alanine Aminotransferase
ANOVA: Analysis of Variance
aOR: Adjusted Odds Ratio
AST: Aspartate Aminotransferase
AUC: Area Under the Curve
BIC: Bayesian Information Criterion
BMI: Body Mass Index
CI: Confidence Interval
EGD: Esophagogastroduodenoscopy
EGJ: Esophagogastric Junction
EWL: Excess Weight Loss
FBG: Fasting Blood Glucose
GEFV: Gastroesophageal Flap Valve
GERD: Gastroesophageal Reflux Disease
GerdQ: Gastroesophageal Reflux Disease Questionnaire
HbA1c: Glycated Hemoglobin
HH: Hiatal Hernia
HR: Hazard Ratio
IQR: Interquartile Range
LA: Los Angeles (Classification)
LCMM: Latent Class Mixed Model
LES: Lower Esophageal Sphincter
LSG: Laparoscopic Sleeve Gastrectomy
MCS: Mental Component Score (SF-36)
MENA: Middle East and North Africa
ML: Machine Learning
OR: Odds Ratio
PAS: Periodic Acid–Schiff
PCS: Physical Component Score (SF-36)
PPI: Proton-Pump Inhibitor
RF: Random Forest
ROC: Receiver Operating Characteristic
RYGB: Roux-en-Y Gastric Bypass
SD: Standard Deviation
SF-36: Short Form (36) Health Survey
STROBE: Strengthening the Reporting of Observational Studies in Epidemiology
T2DM: Type 2 Diabetes Mellitus
ZAP: Z-line Appearance (Classification)
